# Assessment of a numerical model to reproduce event‐scale erosion and deposition distributions in a braided river

**DOI:** 10.1002/2015WR018491

**Published:** 2016-08-27

**Authors:** R. D. Williams, R. Measures, D. M. Hicks, J. Brasington

**Affiliations:** ^1^ School of Geographical and Earth SciencesUniversity of GlasgowGlasgowUK; ^2^ National Institute of Water and Atmospheric ResearchChristchurchNew Zealand; ^3^ School of GeographyQueen Mary, University of LondonLondonUK

**Keywords:** braiding, DEMs of Difference, morphological modeling, Delft3D, terrestrial laser scanning, gravel bed river

## Abstract

Numerical morphological modeling of braided rivers, using a physics‐based approach, is increasingly used as a technique to explore controls on river pattern and, from an applied perspective, to simulate the impact of channel modifications. This paper assesses a depth‐averaged nonuniform sediment model (Delft3D) to predict the morphodynamics of a 2.5 km long reach of the braided Rees River, New Zealand, during a single high‐flow event. Evaluation of model performance primarily focused upon using high‐resolution Digital Elevation Models (DEMs) of Difference, derived from a fusion of terrestrial laser scanning and optical empirical bathymetric mapping, to compare observed and predicted patterns of erosion and deposition and reach‐scale sediment budgets. For the calibrated model, this was supplemented with planform metrics (e.g., braiding intensity). Extensive sensitivity analysis of model functions and parameters was executed, including consideration of numerical scheme for bed load component calculations, hydraulics, bed composition, bed load transport and bed slope effects, bank erosion, and frequency of calculations. Total predicted volumes of erosion and deposition corresponded well to those observed. The difference between predicted and observed volumes of erosion was less than the factor of two that characterizes the accuracy of the Gaeuman et al. bed load transport formula. Grain size distributions were best represented using two *φ* intervals. For unsteady flows, results were sensitive to the morphological time scale factor. The approach of comparing observed and predicted morphological sediment budgets shows the value of using natural experiment data sets for model testing. Sensitivity results are transferable to guide Delft3D applications to other rivers.

## Key Points

- Calibration of morphological model with DEMs of Difference from a natural river
- Validation of Gaeuman et al. mixed grain size bed load formula for reach‐scale application
- Sensitivity of unsteady flow simulations to morphological acceleration factor

## 1 Introduction

Morphological models of braided rivers are used as tools for exploration, explanation, and prediction. A variety of modeling frameworks have been applied to simulate braided river morphodynamics [*Williams et al*., 2016], spanning from reduced‐complexity [e.g., *Murray and Paola*, 1994, 2003] to reductionist approaches [e.g., *Nicholas*, 2013c]. From the perspective of environmental management, particularly applied engineering practice, there is interest in developing morphodynamic models that can be used to support river management decisions [e.g., *Karmaker and Dutta*, 2016]. For example, those concerned with limiting ecosystem degradation and managing flood and geomorphological hazards. Such applications demand models that can simulate three‐dimensional morphodynamics at the reach spatial scale [reach lengths of 10–100 river widths; *Ferguson*, 2007] over annual to centennial time scales. Two‐dimensional (2‐D), physics‐based [sensu *Nicholas*, 2013b] models have potential for simulating qualitative planform characteristics of braided rivers [*Jang and Shimizu*, 2005; *Kleinhans*, 2010; *Lotsari et al*., 2013; *Nicholas*, 2013a, 2013c; *Schuurman et al*., 2013; *Schuurman and Kleinhans*, 2015]. Although morphodynamic modeling with graded sediment has been used in many studies, there has only been limited testing with laboratory data [*Sun et al*., 2015] and in synthetic river settings [*Yang et al*., 2015]. Graded sediment morphodynamic models have not been evaluated using high‐resolution topographic data from a natural river.

### 1.1 Model Assessment Using Natural Experiment Data Sets

In an ideal world, assessment of braided river morphodynamic model predictions would be made using spatially and temporally distributed data on hydrodynamics, sediment flux, and bed level. This would enable the calibration of numerous parameters associated with morphodynamic physics‐based models [*Knight*, 2013; *Church and Ferguson*, 2015]. Unfortunately, logistical and technological constraints limit the feasibility of acquiring such data. With respect to observing bed level change, a revolution in geospatial technologies has enabled the monitoring of fluvial morphology at unprecedented resolution and accuracy [*Carbonneau and Piégay*, 2012; *Tarolli*, 2014; *Passalacqua et al*., 2015]. While the timing of topographic surveys is still constrained to low‐flow conditions, the availability of high‐resolution topography enables the testing of model predictions to extend beyond planimetric comparisons of morphological features, such as riffles, pools, and bars, to posthoc analysis using Digital Elevation Models (DEMs) of Difference (DoD) [*Wheaton et al*., 2010]. A limited number of natural experiment [*Tucker*, 2009] data sets demonstrate the efficacy of using sequences of pre‐event and post‐event DEMs to map morphological change in braided rivers [e.g., *Carrivick et al*., 2012; *Lallias‐Tacon et al*., 2014; *Lane et al*., 2003, 2010; *Milan et al*., 2007; *Moretto et al*., 2014; *Wheaton et al*., 2010; *Williams et al*., 2011]. Such data sets are, however, only useful for the purpose of testing morphodynamic numerical models if a flow record is available during the monitoring period.

### 1.2 2‐D Physics‐Based Modeling

The development of 2‐D numerical morphodynamic models has been challenged by problems associated with the general inaccuracy of sediment transport formulas, the parameterization or exclusion of relevant processes, spatial and temporal discretization, high‐computational overheads of calculations, and the sensitivity of simulation results to small errors in initial and boundary conditions. Investigations have tested steady state shallow‐water predictions of natural braided river hydrodynamics using high‐resolution topographic surveys for boundary conditions, spatially distributed surveys of depth and velocity, and time‐lapse and aerial photography of inundation extent [*Hicks et al*., 2006; *Jowett and Duncan*, 2012; *Nicholas et al*., 2012; *Williams et al*., 2013; *Javernick et al*., 2016].

*Nicholas* [2013a, 2013c] demonstrates how changes to key parameters in a 2‐D morphodynamic model can vary the river style that emerges after centennial‐scale model simulations. This work indicates the potential of a 2‐D physics‐based approach for investigating controls on river pattern but the primary focus was upon simulation of virtual rather than real rivers. The exception to this is a simulation of a braided reach based upon the Waimakariri River [*Nicholas*, 2013c]. Visual comparisons between predicted and natural river planform indicate qualitative similarity but topographic relief is greater than that observed. This was attributed to the assumption of a single grain size and thus a lack of bed armour development. A similar approach of using a braided river's broad characteristics to set up virtual models is used by *Kleinhans* [2010] for the River Rhone and *Crosato and Saleh* [2011] for the Allier River. *Xia et al*. [2013] developed a model of the Lower Yellow River but their analysis of morphological change is restricted to a number of repeat cross‐section surveys. *Lotsari et al*. [2013] simulated 1 year of morphological change along the sand‐bed lower Tana River. Morphological predictions were compared to an annual DoD but the survey data were relatively sparse and considerable scaling was required to match observed volumes of erosion and deposition. In a laboratory environment, *Jang and Shimizu* [2005] demonstrate how topographic survey data can be used to assess model predictions. *Ziliani et al*. [2013] use a reduced‐complexity model to simulate the morphodynamics of the Tagliamento River through multiple flood events. Airborne LiDAR was used for initial topography but this did not include a representation of bed levels in inundated areas, which comprised 30% of braidplain area. Moreover, a repeat topographic survey was not available at the end of the high‐flow event series so evaluation relied solely upon the use of aerial imagery.

The preceding review of applications of morphodynamic models to real rivers indicates that there is considerable scope to enhance model assessment, particularly for models simulating graded sediment. Robust calibration can draw upon a variety of observational data, driven by modeling objectives. For the case of simulating the morphodynamics of natural rivers, model testing needs to consider the affinity between observed and predicted patterns of erosion and deposition. Coupled with this, there is also a need to test whether the correct mass of sediment is being transported at the spatially aggregated reach scale. While observed DoDs provide a new opportunity for performance evaluation, the understanding of parameter sensitivity in graded sediment braided river morphodynamic models is in its infancy. For a sand‐bed river, *Schuurman et al*. [2013] present a sensitivity analysis of a depth‐averaged Delft3D morphodynamic model. Their results showed marginal sensitivity to morphological acceleration and initial and boundary conditions, but showed more sensitivity to bed roughness, sediment transport relation and bed slope effect. To guide future model applications, such analysis is needed for gravel bed braided rivers, where predictions will be sensitive to bank erosion, grain size composition and fractional transport.

### 1.3 Aim and Structure

This paper aims to evaluate the performance of a physics‐based model to predict natural, reach scale, braided river morphodynamics for a single high‐flow event. The following section introduces the study site, observed morphological data, and numerical modeling methodology, including an overview of Delft3D, model setup and the method used to compare observed and predicted morphology. Next, the details and results of a two stage modeling workflow are described: (i) one‐at‐a‐time sensitivity analysis to test model functions and parameters; and (ii) model tuning and assessment of model performance. The final section discusses the parameter sensitivity analysis and the use of natural experiment data for model assessment.

## 2 Study Site and Methodology

### 2.1 Study Site and Observational Data

**Figure 1:**
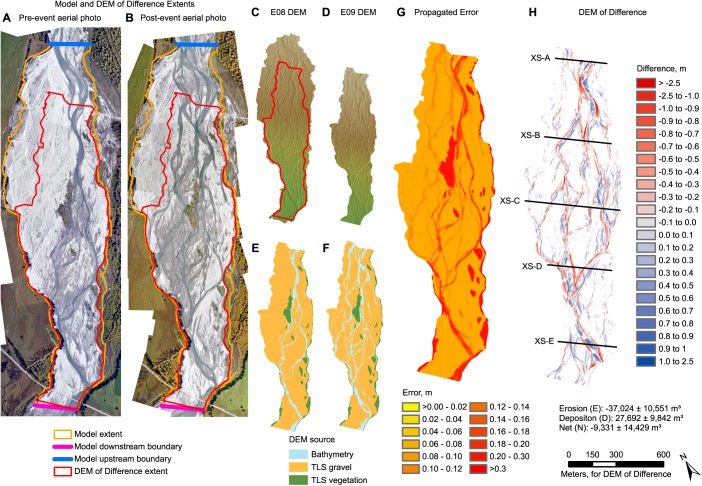
Morphological model domain and DoD. Aerial photos of study area acquired (a) before and (b) after a 227 m^3^ s^−1^ high‐flow event, showing extents of morphological model domain and DoD. DEM acquired (c) before and (d) after the high‐flow event. Note that the post‐storm DEM has a smaller spatial extent than the pre‐storm DEM. Comparisons between the observed and predicted DoDs are thus restricted to the extent of the observed DoD. Techniques used to survey topography for (e) pre‐event and (f) post‐event DEMs. (g) Combined propagated error for probabilistic DoD thresholding at 87% Confidence Interval. (h) DoD showing location of cross sections used to compare observed and predicted bed levels.

This paper uses a natural experiment data set that was acquired as part of the ReesScan project [*Brasington*, 2010; *Williams et al*., 2011]. The 402 km^2^ Rees catchment is located east of New Zealand's Southern Alps and discharges into Lake Wakatipu at 44.85°S, 168.38°E. Catchment geomorphology is described by *Cook et al*. [2014], *Williams et al*. [2013], and *Williams* [2014]. In this paper, the morphodynamics of a 2.5 km long study reach of the Rees River (Figure 1a) are modeled. Hydraulic modeling of this reach is reported by *Williams et al*. [2013]. The reach is characterized by transport‐limited conditions [*Montgomery and Buffington*, 1997] and a longitudinal bed slope of 0.005.

**Figure 2:**
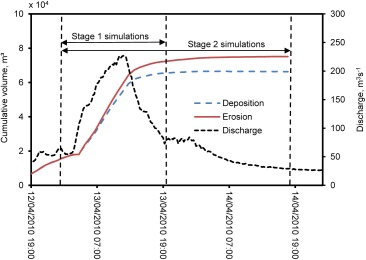
Observed hydrograph, and predicted cumulative erosion and deposition, for simulation of the 227 m^3^ s^−1^ high‐flow event. Horizontal arrows also indicate hydrograph sections used for sensitivity analysis (stage 1) and model tuning (stage 2).

A flow record was derived from a rated gauge 8 km upstream from the study site. During 2010 the mean catchment runoff was 2500 mm. This paper focuses upon simulating a storm event with peak discharge of 227 m^3^ s^−1^ (Figure 2). Aerially exposed and inundated braidplain areas were surveyed before and after the event using a fusion of Terrestrial Laser Scanning (TLS) and optical‐empirical bathymetric mapping [*Williams et al*., 2014, Figure 1]. A DoD was calculated using an error analysis approach that subjected the DEMs to probabilistic thresholding [*Brasington et al*., 2003], using a Confidence Interval of 87%. This approach aims to ensure that DoDs are reliable, by distinguishing between real morphological change and noise. Errors in each DEM were characterized by the Standard Deviation Error associated with the source of elevation data. Constant values were used for three categories: gravelly areas surveyed by TLS; vegetated areas surveyed by TLS; or inundated areas mapped using bathymetric mapping.

**Figure 3:**
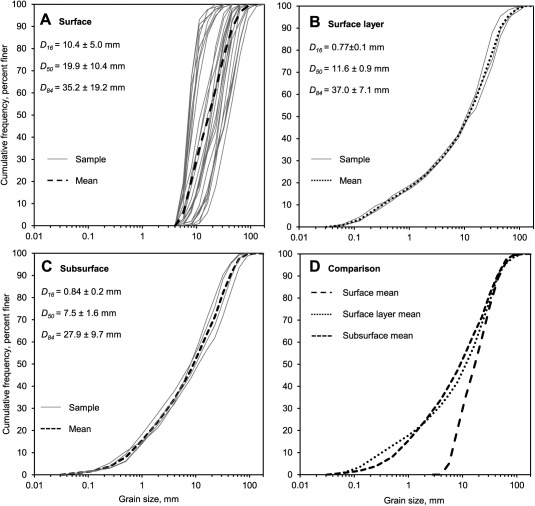
Cumulative grain size distribution curves for (a) surface, (b) surface layer and (c) subsurface sample data, and (d) comparison between the mean cumulative size distributions.

The sedimentology of the study reach was measured by sampling the surface (28 samples), surface layer (3 samples), and subsurface (4 samples; Figure 3). Surface distributions were sampled by means of a 100 clast grid‐count technique that is equivalent to the *Wolman* [1954] pebble count approach. Surface layer sampling followed *Klingeman et al*.'s [1979] and *Klingeman and Emmett*'s [1982] method, where material is removed to the depth of the largest clast on a bar and around a radius equal to 10× the largest clast's long‐axis. Subsurface sampling followed *Church et al*.'s [1987] practical criterion of sieving 100× the weight of the largest surface clast on the bar of interest. The depth of each sample hole was determined by estimating the thickness of the most recently deposited sediment layer. Both volumetric samples were acquired from sites located on active bar surfaces. Particle‐size ranges are described using the terminology of *Blott and Pye* [2001].

### 2.2 Delft3D

Open source Delft3D modeling software (revision 1301, based on release version FLOW4.00.07) was used to simulate depth‐averaged hydrodynamics and morphodynamics. This version incorporates a slope‐based bank erosion algorithm and three bed load transport equations that were coded by the authors; these are elaborated upon below. *Lesser et al*. [2004] and *Deltares* [2011] provide a full description of the hydrodynamic equations and morphodynamic updating scheme, and *Sloff and Mosselman* [2012, and references therein] summarize the model's historical development. The Delft3D modeling system has been widely applied in depth‐averaged mode to simulate interactions between flow, sediment transport, and morphology. The Navier Stokes equations were solved using shallow‐water assumptions and the Boussinesq approximation [*van der Wegen and Roelvink*, 2008]. Shallow‐water equations were solved using an Alternating Direction Implicit method and the horizontal advection terms were spatially discretized using a Cyclic method [*Stelling and Leendertse*, 1992]. The Exner equation was used to calculate changes in bed elevation as a function of net bed load flux in or out of a cell. Since a multi‐grainsize approach was adopted, the Exner equation was applied for each grain size fraction at each morphological time step. The bed was divided into a constant thickness surface active layer which was underlain by underlayers above a nonerodible layer. All sediment in the active layer was assumed to be well mixed and was subject to exchange with sediment in transport [*Hirano*, 1971]. Dissolved and suspended sediment loads were not modeled because bed load is the key control on gravel bed braided river morphology [*Leopold*, 1992].

Two bank erosion schemes were tested: the *ThetSD* scheme that allows the partial redistribution of erosion from a wet cell to adjacent dry cells [*van der Wegen et al*., 2008; *Deltares*, 2011]; and a *repose* scheme that specifies the repose gradient above which slope failure will occur. For the repose scheme, the slope across each cell boundary was calculated, and material was moved downslope in locations where the slope angle exceeds the repose slope. Net slope angle and orientation across each boundary were calculated (including slope parallel to, as well as normal to the cell boundary) to make the scheme relatively independent of grid cell orientation. The flux of material from the active layer was based upon how much the slope is exceeded and the orientation of the slope relative to the cell boundary. Due to the generally low relief of braided rivers, the slope achieved at eroding banks in the model is generally resolution sensitive because the horizontal distance from the base to top of banks in the natural river is generally finer than the resolution of the model.

### 2.3 Model Setup

The extent of the curvilinear model grid is shown in Figure 1a. Gradients in width‐length ratios and cell sizes were minimized, and an automatic orthogonalization procedure was used to reduce deviations from orthogonality of lines between adjacent cell midpoints. The grid was smoothly curved over its length to follow the overall braidplain but no attempt was made to make the cells follow individual braids or topographic features. Grid cells for all simulations, except those that considered grid cell sensitivity, had an average length of 3 m.

The hydrograph recorded at the Invincible gauging station (8 km upstream of the study site) was applied at the upstream flow boundary. At each hydraulic time step, flow was divided between the upstream row of cells using a total discharge boundary, which distributes flow based on local flow depth, cell width, and roughness. Sediment input at the upstream boundary was calculated using the same bed load transport formula that was used to calculate transport across the rest of the model domain. Input at the upstream boundary was therefore equal to the flow's local sediment transport capacity and thus assumes transport‐limited conditions within the reach. Bed level and composition were fixed across the most upstream row of cells. The downstream hydraulic boundary was a water level boundary based upon a rating curve that was constructed by calculating normal depth at the downstream boundary cross section, assuming a longitudinal water slope equal to the mean longitudinal bed gradient and constant bed roughness equal to that set across the model domain. The water level across the downstream boundary was assumed to be horizontal. Bed level change was allowed to occur along the most downstream row of cells but the downstream water level boundary rating curve was not updated. Under conditions of degradation a backwater effect will be caused; under conditions of aggradation artificially low‐flow depths will be predicted. Either way, the downstream boundary discourages morphological change because if bed levels at the boundary lower then flow will deepen and encourage deposition, and vice versa. At the time scale of a single high‐flow event the sensitivity of the hydraulic predictions to relatively small variations in the bed level of the downstream boundary were not considered to be significant. The time step was set to a Courant number less than 10. The minimum depth for sediment transport calculations was set to 0.1 m. Each simulation was initiated with a 2 h long hydraulic spin‐up with no morphological updating to allow the model to generate a stable and realistic hydraulic solution everywhere prior to starting morphological calculations.

### 2.4 Model Assessment

Four approaches were used to compare observed and predicted morphological change. First, a quantitative comparison of total volumes of erosion and deposition, and the net volumetric change, was undertaken for each high‐flow event. The observed morphological change was calculated using a DoD, which was subjected to probabilistic thresholding (section 2.1). Vertical changes in the range from −0.05 to 0.05 m were excluded from the budgets for both observed and predicted morphological change. Second, the area of bed experiencing morphological change was plotted on a histogram. Third, the spatial distribution of erosion and deposition was plotted. The correspondence between the DoDs could then be qualitatively compared; this was particularly useful for comparing simulations during sensitivity analysis. Fourth, cross sections of morphological change were plotted for sensitivity analysis simulations that consider the numerical scheme and bank erosion algorithms. For all comparisons, modeled and predicted changes were calculated at the same spatial resolution.

A one‐at‐a‐time (OAT) approach to sensitivity analysis was adopted so that the results could be easily interpreted and used to guide parameterization of other Delft3d models. Other approaches to sensitivity analysis could, however, be adopted in other applications guided by the results presented here. For example, a sensitivity analysis could be undertaken using an elementary effects (winding stairs) method [*Saltelli and Annoni*, 2010].

Following sensitivity analysis, the model was tuned. In addition to comparing observed and predicted morphological change, this model was also tested by comparing observed and predicted morphological units. This was achieved by running a hydrodynamic simulation using the observed and predicted post‐event DEMs. A flow of 100 m^3^ s^−1^ was used for this testing because this flow yielded the highest braiding intensity, and thus planimetric morphological complexity, across the observed post‐event DEM. The inundation area was classified as wet/dry to define the boundaries of bars. The following metrics were then calculated: channel count braiding intensity [*Howard et al*., 1970]; confluence node density [*Ferguson*, 1993]; and the power relationships between bar perimeter and bar area, bar length and bar width, and bar convex perimeter and bar perimeter [*Kelly*, 2006; *van der Werff and van der Meer*, 2008].

Thirty‐eight simulations were run to produce a baseline model that yielded reasonable morphological predictions (Table 1). This model featured a single active layer and a single underlayer. The assumption of a single underlayer was deemed adequate for simulation of a single high‐flow event where the depth of scour was not expected to exceed the initial thickness of the underlayer. To reduce the computational time for the sensitivity analysis, the baseline model was used to determine the time period when most morphological change occurred (Figure 2). The rate of morphological change decreased significantly once discharge fell to ca. 80 m^3^ s^−1^, so the subsequent sensitivity analysis simulations were stopped at this discharge threshold.

**Table 1:** Model Functions and Parameters Varied in Sensitivity Analysis Experiments^a^

Theme	Experiment	Function/Parameter Examined	Baseline Model	*n*	Description of Sensitivity Runs
Numerical scheme	1	Approach for bed level change calculations	Central scheme	2	Upwind scheme
Hydraulics	2a	Helical flow parameterization	Helical flow parameterization off	2	Helical flow parameterization on
	2b	Bed friction	*k_s_* = 0.04 m	3	*k_s_* = 0.03 and 0.05 m
	2c	Horizontal eddy viscosity	*ν_H_* = 0.1 m^2^ s^−1^	2	*ν_H_* = 1 m^2^ s^−1^
	2d	Discharge	Invincible gauge hydrograph	3	Invincible gauge hydrograph ±15%
	2e	Minimum flow depth	*d*_min_ = 0.10 m	2	*d*_min_ = 0.05 m
Bed composition	3a	Active and under layer thickness	*δ_a_* = 0.25,	4	*δ_a_* = 0.10, 0.50 with *δ_u_* = 2.00 m
			*δ_u_* = 2 m		*δ_a_* = 0.10 m with *δ_u_* = 0.25 m
	3b	Initial bed composition generation (BCG)	No BCG	2	Initial bed composition generation from prior high‐flow event
	3c	Porosity and specific density	*φ* = 0.4, *ρ* = 2600 kg m^−3^	2	*φ* = 0.26, *ρ* = 2732 kg m^−3^
	3d	Sediment mixture	One *φ* intervals based on surface layer and bulk sampling	7	One *φ* intervals based on bulk sampling
					Uniform grain size based on *D*_50_ of bulk sampling
					Division of intervals into sand, gravel, and cobble based on surface layer and bulk sampling
					Finer: one *φ* intervals based on surface layer and bulk sampling with exclusion of very fine sand
					Coarser: all fractions increased by one *φ*, based on surface layer and bulk sampling
					Two *φ* intervals based on surface layer and bulk sampling
Bed load transport	4a	Transport equation	Gaeuman et al.	8	MPM with no *ξ*
					MPM with Egiazaroff *ξ*
					Wilcock and Crowe
					Modified Wilcock and Crowe
	4b	Bed slope effects	Bagnold and Ikeda	3	*α*_bs_ = 0
					Talmon et al.
Bank erosion	5	Repose and simple models	Repose = 0.2	7	No bank erosion
					ThetSD = 0.25, 0.50, 0.75
					Repose = 0.1, 0.3
Calculation frequency	6b	Morphological factor	MorFac = 1	5	MorFac = 2, 5, 10, 20

a *n* = number of simulations undertaken for experiment (including baseline).

## 3 Results

### 3.1 Stage 1: Parameter Sensitivity

#### 3.1.1 Experiment 1: Numerical Scheme for Bed Load Component Calculations

Delft3D makes bed level change calculations at the center of grid cells, but bed load flux components are actually required at points between adjacent grid cells. Experiment 1 tested two numerical discretization schemes, central (used for the baseline model) and upwind, to convert differential equations to algebraic equations that connect discrete nodes on the finite difference grid. Although central schemes are more accurate, since fluxes are less damped, they are also less stable than upwind schemes [cf. *Deltares*, 2011; *Wright*, 2005].

**Figure 4:**
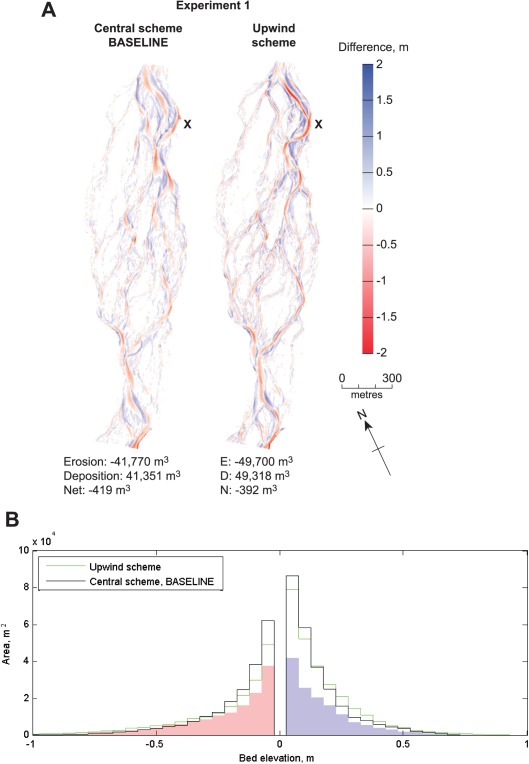
Numerical scheme sensitivity analysis (experiment 1). (a) DoDs. Letters identify areas of interest that are discussed in the text. (b) Sediment budgets. The shaded area on the histogram shows observed morphological change and the lines show model predictions.

Results (Figure 4) showed both schemes over‐predicted volumes of erosion and deposition relative to those observed. Volumes of morphological change for the central scheme were approximately one‐fifth lower than for the upwind scheme and closer to the observed volumes. Units of erosion and deposition were more spatially discrete for the central than the upwind scheme predictions. For example, scour was too deep and elongated for the upwind simulation in the vicinity of X in Figure 4a. The tendency for unrealistically deep scour along primary channels with the upwind scheme was also apparent in cross‐section comparisons (supporting information Figure S1). The histogram for the upwind scheme was characterized by a longer erosional tail attributed to over‐deepening by scour. The distribution of bed elevation changes for the central scheme corresponded more closely to that observed, with similar minima and maxima of elevations of scour and fill. There was a closer correspondence between observed and predicted volumes of erosion than deposition.

#### 3.1.2 Experiment 2: Hydraulics

**Figure 5:**
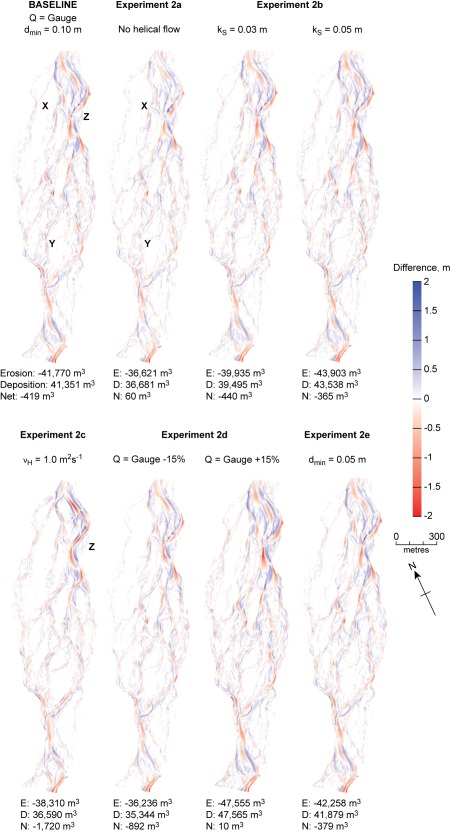
DoDs for hydraulics sensitivity analysis (experiment 2). *k_s_* is Nikuradse roughness length. *ν_H_* is horizontal eddy viscosity. *Q* is discharge. *d_min_* is minimum flow depth. Letters identify areas of interest that are discussed in the text.

Figure 5 shows DoDs for the hydraulics sensitivity experiments. Sediment budget histograms are shown in supporting information Figure S2. Experiment 2a considered the importance of including the effects of helical flow parameterization, which causes the direction of bed load transport to deviate from that predicted by depth‐averaged flow and is important for point bar development [*Dietrich and Smith*, 1983]. The simulation without helical flow parameterization under‐predicted the tail of the erosion distribution. This was because the parameterization of helical flow corrected the direction of bed shear stress compared to that predicted by the depth‐averaged velocity vector, resulting in deeper scour at the outer bends of primary channels and at confluences. Inspection of the DoDs also indicates that patterns of bar development (e.g., in the vicinity of markers X and Y in Figure 5) were more natural for the simulation with a parameterization of helical flow. Thus, inclusion of helical flow parameterization resulted in more realistic confluence and outer bend scour.

Values of Nikuradse roughness length, *k_s_*, horizontal eddy viscosity, *v_H_*, and minimum flow depth, *d_min_*, for the baseline model were set based upon *Williams et al*.'s [2013] hydraulic calibration for the same high‐flow event that is being considered here (Table 1). Simulations for *k_s_* = 0.03 and 0.05 m (experiment 2b) resulted in erosion and deposition volumes decreasing by 4%, and increasing by 5%, respectively, compared to the baseline simulation. Since bed shear stress is estimated by a quadratic friction law, changes in *k_s_* of ±0.01 m had only a relatively minor impact on predicted depth and depth‐averaged velocity, and hence bed shear stress and morphological change.

Predicted volumes of change were relatively sensitive to an order of magnitude increase in eddy viscosity (experiment 2c), with decreases in total erosion and deposition volumes of 8% and 12%, respectively. Predictions for the simulation with higher *v_H_* were associated with a tendency for more longitudinally extensive units of erosion, such as that along the main channel in the vicinity of Z (Figure 5). This resulted in longitudinally simplified channel geometry, with less distinct pool and riffle morphology. These patterns were observed because higher *v_H_* resulted in lower transverse gradients of velocity and more widely distributed bed shear stress. Flows remained competent but bed shear stresses were not as locally intense.

Experiment 2d considered uncertainty in discharge measurement [*Di Baldassarre and Montanari*, 2009; *McMillan et al*., 2010]. There were insufficient data to directly quantify hydrograph uncertainty so a value of 15% was tested, based on *McMillan et al*.'s [2012] benchmark for a similar site. Decreasing discharge by 15% decreased volumes of erosion by 13% and deposition by 15%; increasing discharge resulted in increases of 14% and 15% for erosion and deposition volumes, respectively. The approximately linear relationship between changes in discharge and morphological change occurred because changes in discharge resulted in the expansion or contraction of competent flow [*Ashmore and Sauks*, 2006] through a network of channels across the braidplain that were characterized by similar dimensions. Morphological predictions were relatively insensitive to varying the minimum flow depth from 0.10 to 0.05 m (experiment 2e), with predicted volumes of erosion and deposition varying by 1%. However, varying *d_min_* did not change the threshold depth for sediment transport (*SedThr*), which was kept constant at 0.1 m. Thus, this change had only a minimal impact on predicted bed shear stresses along channels with competent flow.

#### 3.1.3 Experiment 3: Bed Composition

Experiments were executed to investigate the sensitivity of the simulations to active and underlayer thickness (*δ_a_* and *δ_u_*, respectively), initial bed composition, porosity and specific density, and sediment mixture (Table 2 and supporting information Figures S3 and S4). Experiment 3a considered *δ_a_* and *δ_u_*. Morphological predictions showed some sensitivity to active layer thickness, with decreases in *δ_a_* resulting in less morphological change. Decreasing *δ_u_* to 0.25 m curtailed erosion depths to 0.5 m because the base of the model was reached. Inspection of grain size distribution maps for the active layer for each simulation indicated that simulations with a thinner active layer developed a coarser active layer grain size distribution than those with a thicker active layer. This coarsening resulted in higher critical shear stress thresholds for bed load transport and resulted in less morphological change. The thickness of the minimum active layer simulated (0.1 m) is greater than the *D*_84_ grain size (37 ± 7 mm) of the surface layer.

**Table 2:** Predicted Volumes of Morphological Change for Bed Composition (Experiment 3), Bed Load Transport (Experiment 4), Bank Erosion (Experiment 5), Frequency of Calculation (Experiment 6), and Spatial Resolution (Experiment 7) Sensitivity Tests^a^

Experiment	Parameterization	Erosion, m^3^ (% Change From Baseline)	Deposition, m^3^ (% Change From Baseline)	Net (m^3^)
Observed	Not applicable	−37,024 ± 10,551	27,692 ± 9842	−9331 ± 14,429
Baseline	*δ_a_* = 0.25 m, *δ_u_* = 2.00 m	−41,770	41,351	−419
	No initial BCG			
	*φ* = 0.40, *ρ* = 2,600 kg m^−3^			
	1*φ* fractions. a: surface layer, b: bulk			
	BLT: Gaeuman et al.			
	Bed slope: Bagnold and Ikeda (BI)			
	Repose = 0.2			
	MorFac = 1			
	Δ*x* = 3 m			
3a	*δ_a_* = 0.10 m, *δ_u_* = 2.00 m	−40,233 (−4%)	39,308 (−5%)	−926
3a	*δ_a_* = 0.50 m, *δ_u_* = 2.00 m	−43,444 (4%)	43,090 (4%)	−354
3a	*δ_a_* = 0.10 m, *δ_u_* = 0.25 m	−34,105 (−18%)	34,899 (−16%)	794
3b	Initial BCG	−40,233 (−4%)	39,308 (−5%)	−926
3c	*φ* = 0.26, *ρ* = 2732 kg m^−3^	−36,973 (−11%)	36,138 (−13%)	−835
3d	1 *φ* intervals. a: bulk, b: bulk	−43,072 (3%)	42,713 (3%)	−360
3d	Uniform grain size from *D*_50_ bulk	−56,688 (36%)	37,314 (−10%)	−19,374
3d	Three fractions. a: surface layer, b: bulk	−51,859 (24%)	53,182 (29%)	1,323
3d	Finer (no very fine sand). a: adjusted surface layer, b: adjusted bulk.	−40,933 (−2%)	40,142 (−3%)	−791
3d	Coarser (all fractions increased by 1 *φ*). a: adjusted surface layer, b: adjusted bulk	−25,112 (−40%)	23,519 (−43%)	−1593
3d	2 *φ* intervals. a: surface layer, b: bulk	−43,315 (4%)	42,925 (4%)	−390
4a	BLT: MPM, no ξ. Bed slope: BI	−48,735 (17%)	50,095 (21%)	1360
4a	BLT: MPM, Egiazaroff ξ. Bed slope: BI	−39,343 (−6%)	38,528 (−7%)	−815
4a	BLT: Wilcock and Crowe. Bed slope: BI	−42,064 (1%)	42,174 (2%)	110
4a	BLT: Modified Wilcock and Crowe. Bed slope: BI	−42,722 (2%)	42,973 (4%)	250
4b	BLT: Gaeuman et al. Bed slope: no bed slope effect	−49,931 (20%)	49,450 (20%)	−481
4b	BLT: Gaeuman et al. Bed slope: Talmon et al.	−42,717 (2%)	41,898 (1%)	−819
5	No bank erosion	−41,891 (0%)	40,449 (−2%)	−1443
5	ThetSD = 0.25	−42,250 (1%)	40,789 (−1%)	−1462
5	ThetSD = 0.50	−42,473 (2%)	40,841 (−1%)	−1633
5	ThetSD = 0.75	−42,856 (3%)	41,152 (0%)	−1704
5	Repose = 0.1	−39,936 (−4%)	41,489 (0%)	1553
5	Repose = 0.3	−42,494 (2%)	41,388 (0%)	−1106
6	MorFac = 2	−40,891 (−2%)	41,440 (0%)	549
6	MorFac = 5	−41,631 (0%)	40,580 (−2%)	−1050
6	MorFac = 10	−33,545 (−20%)	32,241 (−22%)	−1304
6	MorFac = 20	−20,908 (−50%)	20,114 (−51%)	−794

a The parameterization column describes how parameters were varied from those defined for the baseline model (as listed in Table 1). *δ_a_* is active layer. *δ_u_* is under layer. BCG is bed composition generation. *Φ* is porosity. *ρ* is density. *a* is active layer. *u* is under layer. *φ* is grain size interval (i.e., 1 *φ* refers to a simulations with multiple grain sizes with 1 *φ* size divisions; 2 *φ* refers to a simulations with multiple grain sizes with 2 *φ* size divisions). BLT is bed load transport. MPM is Meyer‐Peter and Müller. *ξ* is hiding and protrusion.

At the start of each baseline simulation, the active layer sediment size distribution was homogeneous across the model domain. In reality, grain size distributions would be spatially variable. To address this assumption, an initial simulation was run to redistribute multiple sediment fractions across the model domain while keeping the bed level fixed (Bed Composition Generation; BCG) [*van der Wegen et al*., 2011]. This was executed using the hydrograph from a precursor event, with a peak of 323 m^3^ s^−1^ (experiment 3b). Including BCG resulted in decreases relative to the baseline of 4% erosion and 5% deposition, and a closer match to the observed erosion histogram tail.

In addition to grain size distribution, sediment porosity and sediment density are needed to calculate sediment transport and bed level change. A porosity of 0.4 is usually assumed [*Mosselman*, 2005] but for mixed gravel and sand sediment compositions, porosity is likely to be lower. *Wu* and *Wang*'s [2006] modification of established porosity formulas yielded a porosity value of 0.26 for the Rees River's surface layer. This was within the range 0.17–0.36 measured for gravel bed rivers by *Milhous* [2001] and *Haschenburger and Roest* [2009]. For density, the baseline simulation used 2600 kg m^−3^ (quartz) but the mean density of schist is 2732 kg m^−3^ [*Tenzer et al*., 2011]. Using physically realistic values for porosity and density (experiment 3c) decreased volumes of erosion and deposition by ca. 11% and 13%, respectively. The predicted change histogram for the revised sediment properties showed better correspondence than the baseline predicted histogram, and the match was particularly good for the tail (<−0.25 m) of the erosion distribution.

During model sensitivity testing, the baseline model used graded sediment, with the active and bed layer initial conditions corresponding to the mean surface layer and subsurface distributions, respectively. Fractions in both layers were divided at one *φ* intervals, ranging from very fine sand to medium boulder. The very fine sand fraction corresponded to the smallest fraction of sediment grains that move as bed load by rolling, sliding, or saltating [*Bridge and Domenicco*, 2008]. A number of experiments were undertaken to test the sensitivity of morphological predictions to the number of discrete sediment fractions simulated and the initial grain size distributions (experiment 3d).

The simulation that used a one *φ* grain size distribution from bulk sampling, for both active and underlayers, resulted in an increase in erosion and deposition relative to the baseline. The simulation with a uniform grain size resulted in deep scour and a relatively long erosion tail in the vertical change histogram. Using three grain classes also resulted in the over‐prediction of morphological change because bed evolution and grain entrainment are overly simplified. Excluding the finest fraction (very fine sand) yielded a small decrease in morphological change. Testing the sensitivity of the predictions by adding one *φ* to the grain size distribution caused considerable under‐prediction of larger magnitude volumetric changes. A final simulation considered the variation due to reducing the number of grain size fractions from one *φ* intervals to two *φ* intervals (from 12 to 6 classes). This resulted in a ca. 50% computational time saving, with only relatively small changes to the overall predicted volumetric change and negligible changes to the DoD. This contrasts to the model with three classes which resulted in greater time savings but unacceptable predictions.

#### 3.1.4 Experiment 4: Bed Load Transport and Bed Slope Effects

**Figure 6:**
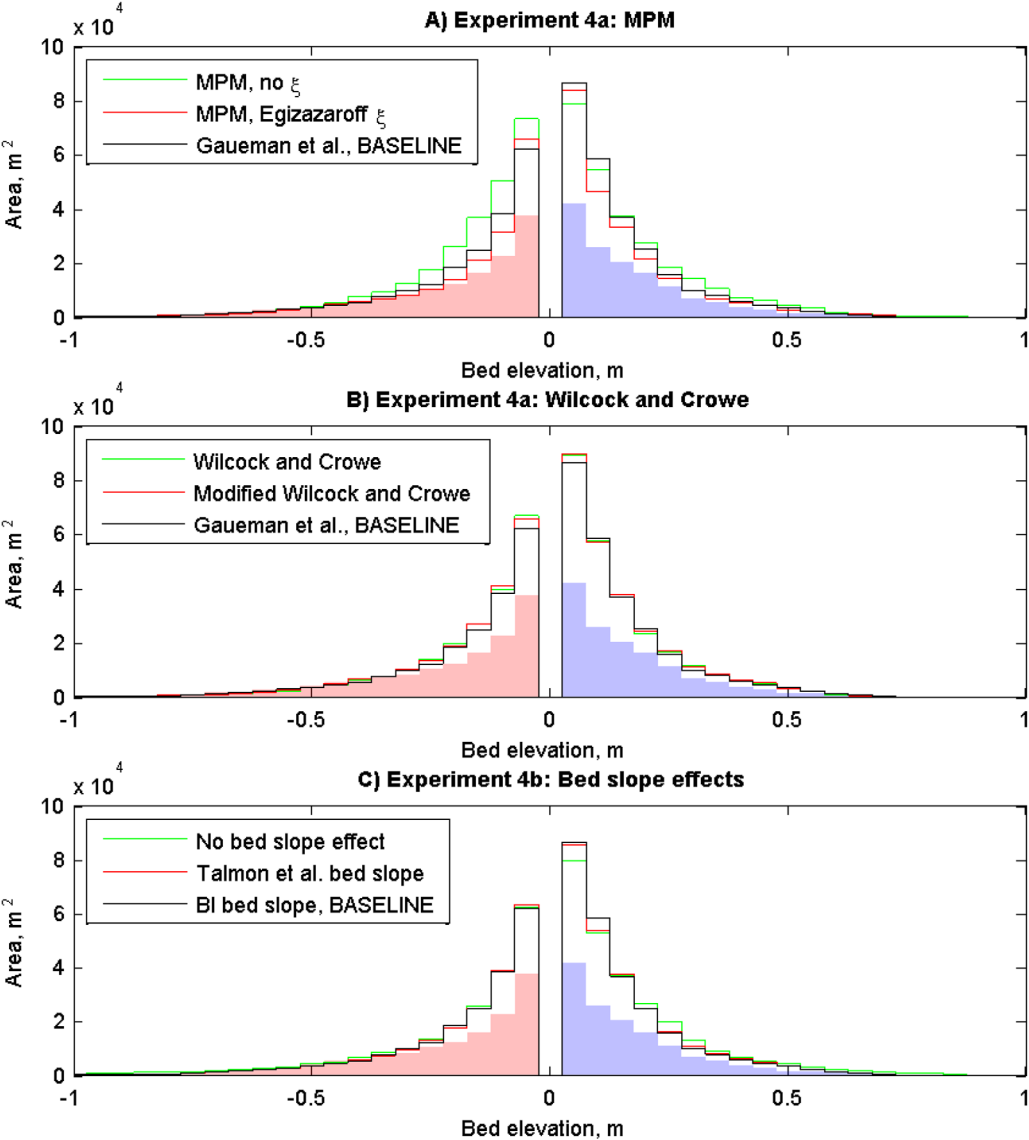
Sediment budgets for bed load transport sensitivity analysis (experiments 4a and 4b). The shaded area on the histogram shows observed morphological change and the lines show model predictions. MPM is Meyer‐Peter and Müller. *ξ* is hiding and protrusion. BI is Bagnold and Ikeda.

*Hicks and Gomez*'s [2005] recommendation of choosing bed load transport formulae on the basis of similarity with the river system upon which they were derived, and comparing the predictions of a number of formulae, was adopted for this paper. Model predictions were relatively sensitive to the choice of bed load transport formula (experiment 4a; Figure 6, Table 2, and supporting information Figure S5). The *Meyer‐Peter and Müller* [MPM; 1948] bed load transport formula, with no hiding and protrusion, over‐predicted erosion. Incorporating the *Egizarazaroff* [1965] hiding and protrusion correction reduced erosion and deposition volumes by approximately one‐quarter. A comparison between DoDs revealed variations in the complexity of change. For example, morphological change in the vicinity of marker X (supporting information Figure S5) was dominated by deeper and more sinuous erosion using the Egiazaroff correction compared to the simulation with no correction. Variations between the *Wilcock and Crowe* [W&C; 2003] and the modified W&C formula [*Gaeuman et al*., 2009] was small. The higher volumetric change associated with the modified W&C formula, relative to the W&C formula, is consistent with the coarsest fractions having greater susceptibility to entrainment. The slightly lower volumetric change associated with the *Gaeuman et al*. [2009] formula, relative to both W&C formulae, was due to the increase in reference shear stress.

The direction of bed load transport deviates from that predicted by depth‐averaged flow due to gravitational forces causing downward acceleration along longitudinal and transverse bed slopes. Experiment 4b tested the parameterization of these bed slope effects (see supporting information for formulae). The simulation with no bed slope effects resulted in greater morphological change and deeper, more longitudinally elongated scour than the simulation that included parameterization of bed slope effects. The simulations that apply bed slope formulations of *Bagnold* [1966], *Ikeda* [1982], and *Talmon et al*. [1995] have similar overall volumes of erosion and deposition, although the simulation that uses Talmon et al.'s formulation was characterized by greater net erosion.

#### 3.1.5 Experiment 5: Bank Erosion

**Figure 7:**
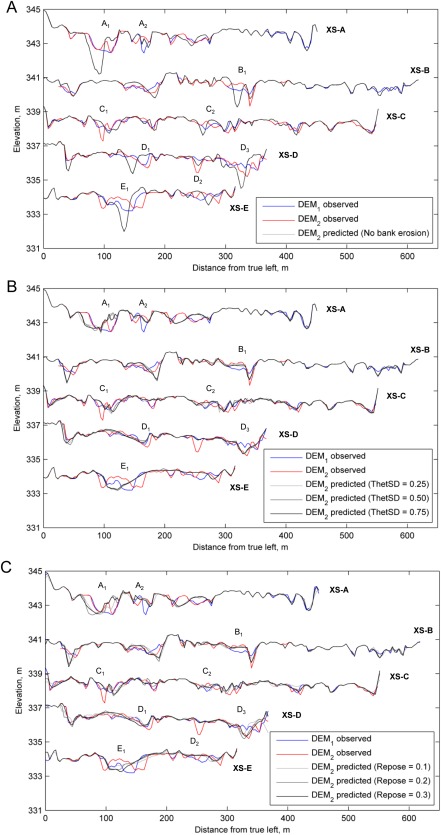
Comparison between surveyed and predicted cross sections levels for bank erosion sensitivity analysis (experiment 5): (a) No bank erosion. (b) ThetSD bank erosion routine. (c) Repose bank erosion routine. Pre‐storm and post‐storm surveys are labeled DEM_1_ and DEM_2_, respectively. Cross sections are located across areas of pertinent morphological change, as indicated in Figure 1h. Letters with numerical subscripts along each cross section are positioned to aid comparison between (a), (b), and (C).

Figure 7 shows five cross sections that compare observed and predicted bed levels for simulations with different bank erosion parametrization. Supporting information Figures S5 and S6 show DoDs and histograms. Table 2 lists sediment budgets. The simulation with no bank erosion was characterized by much deeper scour relative to the other simulations (e.g., A_1_, D_3_, and E_1_ in Figure 7a). Moreover, channels did not erode laterally, resulting in relatively straight channels. Using the ThetSD bank erosion algorithm enabled the erosion of dry cells and thus mitigates the problem of channel overdeepening in simulations with no bank erosion (e.g., A_1_, D_3_, and E_1_ in Figures 7a and 7b). Comparison between the DoDs with no bank erosion and those with the ThetSD routine showed that for simulations with the ThetSD routine the largest channels (e.g., X in supporting information Figure S6) evolved with greater sinuosity. However, the cross‐section plots (Figure 7b) indicate that there was a poor correspondence between the extent of lateral migration predicted by all three ThetSD parameterizations and that observed. For example, lateral channel shifting was in the wrong direction at marker A_1_ and the predicted channel shape at marker E_1_ is considerably different from that observed. From the perspective of the total sediment budget, estimates of erosion and deposition volumes were relatively insensitive to the parameterization of ThetSD.

The repose routine was tested for a range of critical slopes varying from 0.1 to 0.3. The simulation with the shallowest critical slope, 0.1, had the tightest vertical change histogram distribution, with the tail of the erosion distribution closely matching the observed. As the critical slope was steepened, the sinuosity of evolving channels decreased and erosion was deeper. Analysis of the total sediment budget therefore indicated that the repose algorithm performs better than the ThetSD algorithm. Inspection of the predicted cross sections (Figure 7c), however, indicates that disparities remained between the predicted and observed extents of lateral channel migration. At marker A_1_ the repose routine with a critical slope of 0.1 produced a prediction that was closer to the observed channel geometry than the simulations with slopes of 0.2 and 0.3, but lateral migration on the true right of the channel was under‐predicted to a similar magnitude as that observed for the ThetSD routine. At marker E_1_ performance of the repose routine was similar to that predicted by the ThetSD routine.

#### 3.1.6 Experiment 6: Frequency of Morphological Calculations

Morphological change typically occurs on a longer time scale than corresponding changes in flow. Based on this, morphological models typically use a morphological time scale factor, *MorFac*, to speed up morphological changes to a rate where they influence flow dynamics. Existing applications of *MorFac* have focused upon scenarios with uniform sediment and constant or cyclical flow. This has the advantage that the flow inputs can easily be compressed by reducing their duration (in the case of constant flow) or reducing the number of cycles (in the case of cyclic flow). Here the objective was to test the application of *MorFac* to graded sediment, and unsteady flow (experiment 6). In order to implement MorFac with unsteady flow the input flow hydrograph was compressed to reduce its duration in line with the applied scaling factor.

Results (Table 2 and supporting information Figure S7) showed *MorFac* values of 2 and 5 caused some variation in total erosion and deposition volumes but the DoDs remained relatively consistent. Applying *MorFac* values of 10 and 20 resulted in volumetric morphological change decreasing by approximately one‐fifth and one‐half, respectively. The vertical change histogram for a *MorFac* value of 10 showed a slight under‐prediction of erosion. However, erosion and deposition volumes are 80% and 78%, respectively, of the baseline simulation so higher values of *MorFac*, in effect, damped the over‐prediction of morphological change. The simulation with a *MorfFac* value of 20 showed considerable under‐prediction of both erosion and deposition.

### 3.2 Stage 2: Calibration and Assessment

**Figure 8:**
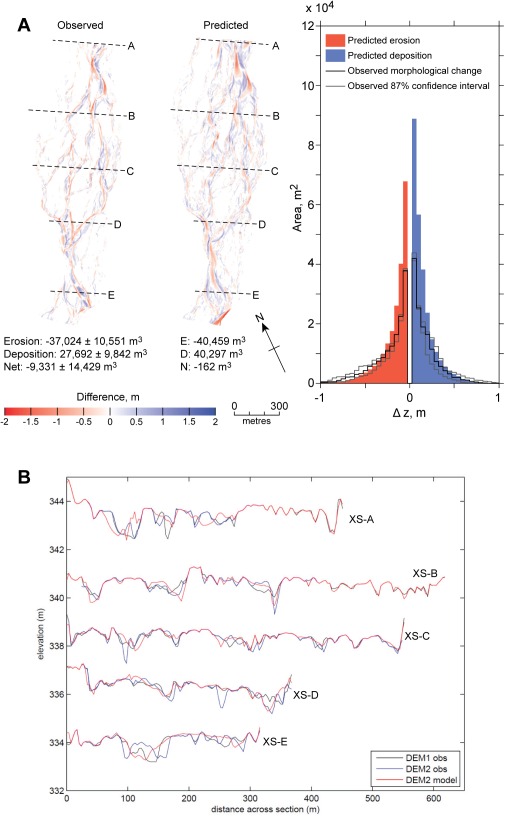
(a) Observed and predicted DoDs and sediment budget for calibrated model of 227 m^3^ s^−1^ event. (B) Comparison of observed and simulated cross sections (stage 2) of the 227 m^3^ s^−1^ event. Pre‐storm and post‐storm surveys are labeled DEM_1_ and DEM_2_, respectively. Cross sections are located across areas of pertinent morphological change, as indicated in Figure 1h.

Results from the sensitivity analysis were used for model tuning. A further set of simulations was executed that combined the best performing parameterizations from each sensitivity experiment. These simulations were executed for an additional 24 h so that flows receded to 29 m^3^ s^−1^ at the end of the simulation (Figure 2). The calibrated model (Table 3 and supporting information movie S1) showed strong spatial coherence between units of observed and predicted morphological change (Figure 8). The tails of distribution of predicted and observed erosion and deposition volumes were similar. The predicted distribution was, however, more leptokurtic than the observed distribution. Predicted volumes of erosion and deposition were both greater than observed. The total erosion volume predicted (40,459 m^3^) was within the observed 87% confidence interval (−37,024 ± 10,551 m^3^) but the total deposition volume predicted (40,297 m^3^) was greater than the observed 87% confidence interval (27,692 ± 9842 m^3^). A comparison between the observed and predicted post‐event cross sections (Figure 8) shows that morphological change occurred in similar parts of the braidplain. It also shows the dimensions of observed and predicted channels were similar. In particular, the model did not over‐predict the depth of scour.

**Table 3:** Model Functions and Values of Parameters Used in Calibrated Model of 227 m^3^ s^−1^ Event

Function/Parameter	Value
Numerical scheme for bed level change calculations	Central
Helical flow parameterization	On
Bed friction	*k_s_* = 0.05 m
Horizontal eddy viscosity	*ν_H_* = 0.1 m^2^ s^−1^
Discharge	Invincible gauge hydrograph
Minimum flow depth	*d*_min_ = 0.05 m
Threshold depth for sediment calculations	*SedThr* = 0.1 m
Active layer thickness	*δ_a_* = 0.25 m
Under layer thickness	*δ_u_* = 2 m
Initial bed composition generation	Generated from precursor storm event
Porosity	*Φ* = 0.26
Specific density	*ρ* = 2732 kg m^−3^
Sediment mixture (active layer)	One *φ* intervals based on surface layer sampling
Sediment mixture (under layer)	One *φ* intervals based on bulk sampling
Bed‐material transport equation	*Gaeuman et al*. [2009]
Bed slope effects	*Talmon et al*. [1995]
Bank erosion model	*Repose* = 0.2
Hydraulic time step	Δ*t* = 0.6 s
Morphological factor	*MorFac* = 5
Grid resolution	Δ*x* = 3 m

Table 4 lists the results from metrics that compare the observed and predicted planimetric morphology of the study reach. Braiding intensity and confluence node density were predicted to be slightly lower after the high‐flow event, indicating some network simplification. Metrics for the ratio between bar perimeter and bar area, and bar convex perimeter and bar perimeter were similar for both observations and predictions. The ratio between bar length and bar width was predicted to be lower than that observed.

**Table 4:** Metrics Used to Assess Calibrated Model Performance^a^

Metric		Observed	Predicted
Braiding intensity		6.8	6.4
Confluence node density, nodes km^−2^		91	83
Least squares regression coefficient of determination, *R* ^2^, for relationship between:	Bar perimeter and bar area	0.986	0.985
	Bar length and bar width	0.903	0.881
	Bar convex perimeter	0.999	0.999

a Values were calculated from running a 100 m^3^ s^−1^ steady state hydrodynamic simulation across observed and predicted DEMs.

## 4 Discussion

### 4.1 Parameter Sensitivity analysis

An unsteady model of braided river morphodynamics was developed by starting with a sensitivity analysis, followed by model tuning and assessment. This section discusses findings from the sensitivity analysis, focusing upon important findings relating to bed composition, bed load transport, bank erosion, and the frequency of morphological calculations.

#### 4.1.1 Bed Composition

The sensitivity analysis demonstrated the importance of representing graded sediment in the model. *Nicholas* [2013c] suggested that his physics‐based model of the Waimakariri had a tendency to generate cross‐sectional relief with a greater range than that observed because only a single grain size was represented in the model. This effect was confirmed by the single grain size model for the Rees, with erosion occurring to the bed of the underlayer at confluences and toward the outer bank of primary channel bends. Multiple grain size fractions are necessary to model armour layer formation; a ubiquitous feature of gravel bed rivers [*Yager and Schott*, 2013]. Results suggest two *φ* intervals are adequate for representing grain size distribution, and representing the active layer with a slightly coarser grain size distribution is appropriate.

Existing guidance suggests *δ_a_* should equal *D*_84_ grain size for graded sediment or half the bed form height for uniform sediment [*Mosselman*, 2012]. *Sloff and Mosselman* [2012], however, found that it was necessary to use *δ_a_* = 1 m for a graded sediment simulation of the River Rhine's bifurcation, which was 10 times thicker than half the dune height measured in the field. *Nicholas* [2013c] used *δ_a_* = 1 m for a uniform grain size simulation. Sensitivity analysis results from the Rees modeling shows that morphological predictions are sensitive to *δ_a_* due to feedback associated with bed armouring. The calibrated model used *δ_a_* = 0.25 m; a factor of 9 greater than subsurface *D*_84_. The use of an initial BCG run to set a spatially variable initial grain size distribution in the active layer yielded smaller changes in bed load transport than observed by *van der Wegen et al*. [2011] for a simulation of San Pablo Bay, California. This is likely because the San Pablo Bay model used a constant composition for the active and underlayers whereas for the Rees model the initial active and underlayer sediment compositions were different (from surface layer and bulk sediment sampling). The initial active layer of the Rees model is therefore already better defined than that in the San Pablo Bay model.

#### 4.1.2 Bed load Transport and Bank Erosion

Transport of loosely consolidated sand and gravel bed load in the Rees River is transport‐limited. Entrainment is thus controlled by flow intensity, which is expressed in empirical bed load transport formulae in terms of shear stress on the river bed [*Warburton*, 2011]. The sensitivity testing of bed load transport formulae demonstrated the superior performance of formulas that included the effects of hiding and protrusion. The MPM formula, with no hiding and protrusion, over‐predicted erosion as a consequence of the formula assuming equal mobility. The tendency for this bed load transport formula to cause deep scour has also been noted by *Kleinhans et al*. [2008]. Similar performance was obtained from the MPM formula with the addition of hiding and protrusion effects and the W&C formula, and associated variants, which were directly developed for predicting the transport of graded sediments. Comparative results, albeit using different nonuniform transport formulas, have been obtained from numerical model simulations of laboratory bends [*Fischer‐Antze et al*., 2009; *Feurich and Olsen*, 2011]. Overall, it is salient to note that, relative to the observed DoD, volumes from the Gaeuman et al. predictions over‐estimated erosion by 26% and over‐estimated deposition by 49%. It is notable that the magnitude of this difference is greater than the differences between the different transport formulae. However, this should be interpreted in the context of typical bed load transport equation performance, where predictions are estimated to be within a factor of two about two‐thirds of the time [*Ackers and White*, 1973; *Gomez and Church*, 1989]. The total volumes of erosion and deposition, and the overall sediment balance of the monitored reach, are likely strongly influenced by sediment supply at the upstream boundary. All the modeling presented here used an equilibrium upstream boundary condition for sediment and it is possible that by changing this a better fit with observed data could have been achieved. For example, by reducing sediment input it is likely that overall deposition volumes could have been reduced to better fit the observed data. The sediment balance may also be influenced by local transport‐limited sediment transport in each grid cell. Accounting for a spatial lag between bed shear stress and entrainment, transport, and deposition of sediment may therefore be important. The Gaeuman et al. formula has the best physical basis for predicting sediment similar to the Rees. At the reach scale, the comparison of morphological change estimated using DoDs to those predicted by numerical modeling contributes to validating the Gaeuman et al. formula. Importantly, model predictions of low magnitude vertical change provide insight into morphodynamics that would be thresholded out from observations.

With respect to bank erosion, both the ThetSD and repose bank erosion routines were effective at preventing the channel over‐deepening which prevailed without any bank erosion routine. Predictions were more sensitive to changes in the values of the repose angle than the proportion of sediment transferred from dry to wet cells using the ThetSD routine. This is because the repose angle is applied to neighboring cells that are both wet‐wet and wet‐dry during each morphological time step, whereas the ThetSD routine is only applied to neighboring cells that are wet‐dry. At low flows, when the bank is aerially exposed, bank erosion has been observed along the Rees River's braided channel network [*Rennie*, 2012; *Williams et al*., 2015]. The exact dynamics of erosion of sharp banks at high flows, when the banks of bars are often inundated by shallow water, has not been directly monitored. It is likely, however, that inundated banks will continue to erode. The Repose routine is thus considered a more physically plausible routine than the ThetSD routine but it does result in averaged bed and bank slopes. The lateral migration rates that are predicted by both the Repose and ThetSD routines do not match those observed. In some locations this could be due to propagation of small errors in flow patterns, for example, flow splits at diffluences, but it is also likely that both bank erosion routines are overly simplified and miss key factors affecting erosion rate.

A fundamental improvement to numerical bank erosion schemes would be to make them independent of grid resolution and orientation. Morphological modeling of braided rivers is generally conducted at a resolution where bank height is similar or less than grid cell resolution, meaning that bank slope is not well represented by the model grid. Bank height and bank‐toe transport rate may thus be more suitable parameters than bank slope for use in any bank erosion routine as they are likely to be less sensitive to cell resolution and orientation. Subgrid scale representation of banks (split cells) is one approach which solves resolution related issues but it presents other difficulties including increased computational time and difficulty in locating banks, especially during the drying and wetting of bar tops. In the Rees setting where the braid banks are composed largely of cohesionless fine gravel and sand, incorporating the effects or transient bank‐toe protection by blocks of failed cohesive bank material (e.g., as applied by *Ahasi et al*. [2013]) is unlikely to significantly influence the bank retreat process.

#### 4.1.3 Frequency of Morphological Calculations

The results from the sensitivity analysis indicate that the predictions are more sensitive to *MorFac* than has been reported elsewhere. For example, *Nicholas* [2013c] and *Kleinhans et al*. [2008] reported that scaling factors of 100 and 200, respectively, have little discernible impact on constant discharge or block‐hydrographs. Here the results from unsteady discharge simulations indicate that a scaling factor of 20 results in significant under‐prediction of morphological change. The most likely explanation is that increasing *MorFac* causes the input flow hydrograph to be compressed. The compressed hydrograph has steeper rising and falling limbs. Flow is therefore less likely to attenuate over the length of the reach. A more physically realistic approach to morphological acceleration might be to retain the same hydrograph and hydraulic time step but to make morphological calculations only on intermittent hydraulic time steps. This would not result in as big a timesaving as the currently implemented *MorFac* approach available in Delft3D because it only reduces the effort involved in morphological calculation rather than the effort in the hydraulic calculation as well. It would however have the advantage that the hydraulics would not be compromised. This is particularly important for simulation of the rapid variations in flow experienced during floods.

#### 4.1.4 Natural Experiment Data for Model Assessment

The testing of the Delft3D model to determine the capability of the model to predict bed level change for a single high‐flow event is a step toward testing model predictions of natural river morphodynamics over longer time series. It is acknowledged that the morphological evolution of braided rivers is sensitive to initial conditions [*Lane and Richards*, 1997], both in the field and when modeling. Predictions of morphological change at the scale of individual high‐flow events are, however, likely to be characterized by less uncertainty than predictions of morphological change forced by multiple high‐flow events. A single high‐flow event assessment of topographic change is thus appropriate in this context.

There are only a limited number of natural experiment data sets that record the morphodynamics of braided rivers through a series of high‐flow events. The Rees River DoDs are characterized by low vertical error compared to other natural experiment data sets, and are also supplemented by a continuous flow record. Following appropriate probabilistic thresholding, the DoDs have been shown to be suitable for morphodynamic model sensitivity testing. A remaining challenge for evaluating morphodynamic models with a DoD approach is to address the unknown flux of sediment in and out of the study reach from the upstream and downstream boundaries respectively. While this flux can be measured by bed load sampling or the estimation of sediment step lengths, the uncertainty associated with fluxes into and out of the reach remains a primary source of error associated with using DoDs to assess morphological model predictions. Transport‐limited upstream boundary conditions are assumed for the modeling reported here. Longer term simulation may, however, need to use an upstream boundary condition where bed level is not fixed and sediment input is not equal to the flow's local sediment transport capacity. Specification of nonequilibrium upstream boundary conditions will require consideration of sediment supply rate and composition, and how these vary across the width of the boundary and over time.

## 5 Conclusion

This study showed that a numerical model reproduced important features of bed level change in a braided river. Predictions of the location and total volume of erosion and deposition corresponded well to those observed. At the reach scale, morphological predictions validated the use of the Gaeuman et al. mixed grain size bed load transport formula; the difference between predicted and observed volumes of erosion was less than the factor of two that typically characterizes the accuracy of such predictions. Through a sensitivity analysis, observed and predicted DoDs were used to test model parameterization. Observed and predicted planimetric metrics, such as braiding intensity and bar shape, were also used to gain further insight into the performance of the model. While there are many ways to carry out a sensitivity analysis and calibration of a morphological model, the results and approach reported in this investigation are transferable to guide physics‐based model applications to other rivers.

Results indicate that future model development efforts should be directed toward improving the realism of bank erosion processes in the model. In particular, the bank erosion scheme needs to be made independent of grid resolution and orientation. Such efforts need to be coupled with the application of suitable metrics to test whether lateral erosion rates are realistic and whether bed load transport pathways connect observed zones of erosion and deposition. There is also a need to test the sensitivity of the model to the upstream sediment boundary condition and to develop an appropriate framework for applying high morphological acceleration factors in unsteady flow simulations. The simulation of multiple high‐flow events would provide further insight into model performance. Such modeling needs to be underpinned by the development of appropriate metrics that exploit the information in high‐resolution terrain data to enable the comparison of observed and predicted topography.

## Supporting information

Supporting Information S1

Movie S1
