# Supplementary material for: Assessment of a numerical model to reproduce event‐scale erosion and deposition distributions in a braided river
Source: Water Resour Res. 2016 Aug 27;52(8):6621–42. doi: 10.1002/2015WR018491 (PMC5042110; doi:10.1002/2015WR018491)
Supplement: Supplementary file 1 — Supporting Information S1 [file WRCR-52-6621-s001.doc]

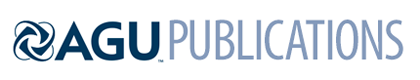


*Water Resources Research*

Supporting Information for

**Assessment of a braided river numerical model to reproduce event-scale erosion and deposition distributions**

R.D. Williams^1^, R. Measures^2^, M. Hicks^2^, J. Brasington^3^

^1^School of Geographical and Earth Sciences, University of Glasgow, Main Building, East Quadrangle, G12 8QQ, United Kingdom, richard.williams@glasgow.ac.uk.

^2^National Institute of Water and Atmospheric Research, PO Box 8602, Christchurch, 8011, New Zealand.

^3^School of Geography, Queen Mary, University of London, London, E1 4NS, United Kingdom.

**Contents of this file**

Text S1

Figures S1 to S8

Movie M1

**Introduction**

This supplementary material provides supplementary text (Text S1) on numerical schemes for bedload component calculations, and presents the bedload transport and bed slope effects formulae that were used in model simulations. It also includes a number of supplementary results figures (Figures S1 to S8). A separate animation file (2015WR018491-M1.avi) shows a paired sequence of depth and cumulative morphological change maps for the calibrated 227 m^3^s^-1^ event simulation.

Text S1. Supplementary text on methods

***Numerical scheme for bedload component calculations***

Drawing on *Wright* [2005] and *Wu* [2008], the difference between upwind and central schemes can be conceptualized by considering a one-dimensional finite difference grid, where a point, *x_i_*, is addressed relative to the position of its neighbors (i.e. x*_i-1_, x_i+1_*)*.* Using an upwind difference scheme the first order derivative of a function, *f,* can be approximated at a point *x_i_* as:

$\left(\frac{\partial f}{\partial x}\right)_{i}\approx\frac{f_{i}-f_{i-1}}{\Delta x}$ (1)

The corresponding central difference scheme is:

$\left(\frac{\partial f}{\partial x}\right)_{i}\approx\frac{f_{i+1}-f_{i-1}}{2\Delta x}$ (2)

The upwind scheme’s use of points *x_i_* and *x_i-1_* means the bedload flux between adjacent cells is set to the value at the water level point that is in an upwind direction relative to the net transport direction. The central approach uses values positioned around point *x_i_*. This results in net transport direction not being considered.

***Empirical bedload transport formulae***

*Meyer-Peter and Müller (1948)*

The *Meyer-Peter and Müller* [MPM; 1948] relation was developed to predict sediment transport in alpine streams in Switzerland. The formulae were developed by measuring the transport of well sorted fine gravel in laboratory conditions:

$q^{*}=8\left(\tau^{*}-\tau_{c}^{*}\right)^{1.5}$ (3)

where $q^{*}$ is the dimensionless transport rate, $\tau^{*}$ is dimensionless shear stress and $\tau_{c}^{*}$ is dimensionless critical shear stress, which Meyer-Peter and Müller estimated to be 0.047. Whilst the relation was developed for a universal grain size, it can be implemented in Delft3d using the mean grain size of the sediment fraction being considered and a hiding function to account for the effects of hiding and protrusion.

The entrainment of particles from sediment mixtures is influenced by the shielding of fine particles by coarse particles (hiding), and increases in exposure of large grains to the main flow, relative to homogeneously sized sediments [protrusion; *Einstein*, 1950]; the effects of hiding and protrusion. The sensitivity of the MPM bedload transport relation is tested using no hiding and protrusion correction and the *Egiazaroff* [1965] formulation:

$\xi=\left(\frac{{}^{10}\log19}{{}^{10}\log19+{}^{10}\log\left(D_{i}/D_{50}\right)}\right)^{2}$ (4)

where $\xi$ is the hiding and protrusion correction, *D_i_* and *D_50_* are the characteristic grain size of the *i*th size range and the median grain size respectively. The Egiazaroff formulation tends towards equalisation of mobility for a sediment mixture but for very coarse grains in a mix the deviation is more towards size independent entrainment [*Parker*, 2008].

*Wilcock and Crowe (2003)*

Use of the MPM relation to predict sediment transport rates in gravel-bed rivers has largely been superseded by other formulae that consider multiple particle size fractions and that include the effects of hiding and protrusion [*Wilcock et al.*, 2009]. *Wilcock and Crowe’s* [2003] relation makes use of flume experiments reported in *Wilcock et al.* [2001]. These experiments considered the transport of sand and gravel mixtures, with varying sand content, and demonstrated that increases in sand proportion caused disproportionate increases in gravel transport. The steps associated with calculating bed material transport using the relation of Wilcock and Crowe are summarised below, drawing upon the calculation procedures outlined in *Parker* [2008].

First, the user must specify grain sizes and fractions of the surface layer (*D_i_,F_i_*). Next, the dimensionless submerged specific gravity of the sediment, *R_g_,* is calculated:

$R_{g}=\frac{\rho_{s}-\rho_{f}}{\rho_{f}}$ (5)

where *ρ_f_* and *ρ_s_* are the densities of fluid and sediment respectively. The surface geometric mean size, *D_g_*, is then calculated and a dimensionless Shields number, $\tau_{sg}^{*}$ is computed from the following:

$\tau_{sg}^{*}=\frac{\tau_{sb}}{\rho_{f}R_{g}gD_{g}}$ (6)

This estimate of the dimensionless Shields number is then used to calculate the shear velocity associated with skin friction, $u_{*s}$:

$u_{*s}=\sqrt{\frac{\tau_{sb}}{\rho_{f}}}$ (7)

where $\tau_{sb}$ is boundary shear stress due to skin friction at the bed. The values of the dimensionless bed load transport parameter, $W_{i}^{*}$ and the volumetric transport rate of bedload per unit width of the *i*th size range, *q_i_*, are then calculated:

$W_{i}^{*}=\frac{R_{g}gq_{i}}{F_{i}u_{*s}^{3}}$ (8)

with the aid of:

$W_{i}^{*}=0.002\Phi^{7.5}$for $\Phi<1.35$ (9)

$W_{i}^{*}=14\left(1-\frac{0.894}{\Phi^{0.5}}\right)^{4.5}$ for $\Phi\geq1.35$ (10)

$\Phi=\frac{\tau_{sg}^{*}}{\tau_{ssrg}^{*}}\left(\frac{D_{i}}{D_{g}}\right)^{-b}$ (11)

$\tau_{ssrg}^{*}=0.021+0.015\exp\left(-20F_{i}\right)$ (12)

$b=\frac{0.67}{1+\exp\left(1.5-\frac{D_{i}}{D_{g}}\right)}$ (13)

where *F_i_* is the fraction of the surface layer material that is sand and $\tau_{ssrg}$ is the dimensionless reference shear stress for the geometric mean particle size on the bed surface. Equation 11 is a “hiding function” and the value of exponent *b* expresses the degree to which equal mobility is achieved during particle entrainment and transport processes. The total volumetric bedload transport rate per unit width, $q_{\Gamma}$ can then be calculated:

$q_{\Gamma}=\sum_{i=1}^{n} q_{i}$ (14)

and the mass fraction of material of the *i*th grain size range in the bedload can then be computed:

$f_{bi}=\frac{q_{i}}{\sum_{i=1}^{n} q_{i}}$ (15)

*Modified Wilcock and Crowe (2003)*

Gaeuman *et al.* [2009] modified the description of the bed surface condition in the Wilcock and Crowe model. They replaced *F_i_* with the arithmetic standard deviation of grain size on the phi scale (*σ_φ_*) as a measure of the bed surface condition to calculate reference shear stress. The model is calibrated to the experimental data used in the derivation of the Wilcock and Crowe model. The calculation of *σ_φ_* and the revised formulae for calculating b and $\tau_{ssrg}$ are:

(16)

$b=\frac{1-\alpha_{0}}{1+\exp\left(1.5-\frac{D_{i}}{D_{g}}\right)}$ (17)

$\tau_{ssrg}^{*}=\theta_{c0}+\frac{0.0365-0.021}{1+\exp\left(10.1\left(\sigma_{sg}-1.4\right)\right)}$ (18)

where $\theta_{c0}$ and *α_0_* are user specified parameters, and take values of 0.021 and 0.33 respectively when calibrated to the Wilcock and Crowe laboratory data. Note that the latest Delft3d user manual [*Deltares,* 2014] refers to this bedload transport relation as the *Gaeuman et al.* [2009] laboratory calibration.

*Gaeuman et al. (2009)*

The modified Wilcock and Crowe relation was tested by *Gaeuman et al.* [2009] using data from bed load sampling in the Trinity River, California. The mean surface grain size at the Trinity River sampling locations ranged from 0.038 to 0.058 m, and samples were obtained during several dam releases that created peak discharges of approximately 300 and 150 m^3^s^-1^. Due to the presence of a dam upstream of the sampling sites, the transport conditions were sediment supply limited. Under these field conditions, the Wilcock and Crowe equations predicted total bed load transport rates within a factor of 2 of sampled rates, for 68% of samples. The equations performed well for finer fractions in the surface layer material but they consistently under predicted the transport of large cobbles (128-256 mm), often by more than an order of magnitude. Calibration to the Trinity River dataset yielded revised implementations for the calculation of b and $\tau_{ssrg}$:

$b=\frac{1-\alpha_{0}}{1+\exp\left(1.9-\frac{D_{i}}{3D_{g}}\right)}$ (19)

$\tau_{ssrg}^{*}=\theta_{c0}+\frac{0.052-0.03}{1+\exp\left(7.1\left(\sigma_{sg}-1.66\right)\right)}$ (20)

where $\theta_{c0}$ and *α_0_* take values of 0.03 and 0.33 respectively. Note that the latest Delft3d user manual [*Deltares,* 2014] refers to this bedload transport relation as the *Gaeuman et al.* [2009] Trinity River calibration.

***Bed slope effects formulae***

In addition to secondary circulation, the direction of bedload transport also deviates from that predicted by depth-averaged flow due to gravitational forces causing downward acceleration along longitudinal and transverse bed slopes:

$\overrightarrow{S'_{b}}=\alpha_{s}\overrightarrow{S''_{b}}$ (21)

where $\overrightarrow{S'_{b}}$ is the unadjusted bedload transport vector, $\overrightarrow{S''_{b}}$ is the magnitude of bedload transport vector, and *α_s_* is a coefficient. The effect of bed slope on bedload transport can be considered theoretically by balancing forces, and assuming a smooth bed with no bed forms, for longitudinal [*Bagnold*, 1966] and transverse [*Ikeda*, 1982] slopes:

(22)

where *α_bs_* is a tuning parameter, set here to 1, *ø* is slope angle, *z* is elevation and *s* is a reference coordinate length. The reader is referred to *Deltares* [2011] for the subsequent formulation of the bedload vector.

An alternative to this theoretical derivation is *Talmon et al.’s* [1995] extension of *Koch and Flokstra’s* [1981] formulation, which based upon empirical laboratory experiments, and is:

$\alpha_{s}=1+\alpha_{bs}\left(\frac{\partial z}{\partial s}\right)$ (23)

The bedload vector is then calculated from:

(24)

where and are final and original directions of sediment transport and $f(\theta)$ is calculated from:

$f(\theta)=A_{sh}\theta_{i}^{B_{sh}}\left(\frac{D_{i}}{H}\right)^{C_{sh}}$ (25)

where *A_sh_*, *B_sh_* and *C_sh_* are tuning coefficients, set here to values of 9, 0.5 and 0.3 respectively, based on *Talmon et al.* [1995].

**References**

Bagnold, R. A. (1966), An approach to the sediment transport problem from general physics, 42 pp., Washington, DC.

Deltares (2011), *Delft3d-FLOW Simulation of multi-dimensional hydrodynamic ﬂows and transport phenomena, including sediments: User Manual*, a documentation report, 688 pp., Deltares, Delft, The Netherlands.

Deltares (2014), *Delft3d-FLOW Simulation of multi-dimensional hydrodynamic ﬂows and transport phenomena, including sediments: User Manual*, a documentation report, 710 pp., Deltares, Delft, The Netherlands.

Egiazaroff, I. V. (1965), Calculation of non-uniform sediment concentrations, *Journal of Hydraulic Engineering*, *91*(4), 225-248.

Gaeuman, D., E. D. Andrews, A. Krause, and W. Smith (2009), Predicting fractional bed load transport rates: Application of the Wilcock-Crowe equations to a regulated gravel bed river, *Water Resources Research*, *45*(6), W06409, doi:10.1029/2008wr007320.

Ikeda, S. (1982), Incipient motion of sand particles on side slopes, *Journal of Hydraulics Division, ASCE*, 108(1), 95-114.

Koch, F. G., and C. Flokstra (1981), Bed level computations for curved alluvial channels, in *Proceedings of the 19th Congress IAHR*, New Delhi, India, February 1981.

Meyer-Peter, E., and R. Müller (1948), Formulas for bedload transport, Proceedings of the 2nd meeting of the International Association for Hydraulic Research, pp. 39-64, International Association of Hydraulic Engineering and Research, Stockholm, Sweden, 6 July 1948.

Parker, G. (1990), Surface-based bedload transport relation for gravel rivers, *Journal of Hydraulic Research*, *28*, 417-436.

Parker, G. (2008), Transport of gravel and sediment mixtures, in *Sedimentation Engineering*, edited by J. J. G. Garcia, American Society of Civil Engineers, Reston, VA.

Talmon, A. M., N. Struiksma, and M. C. L. M. Van Mierlo (1995), Laboratory measurements of the direction of sediment transport on transverse alluvial-bed slopes, *Journal of Hydraulic Research*, *33*(4), 495-517, doi:10.1080/00221689509498657.

Wilcock, P. R. (2001), Toward a practical method for estimating sediment-transport rates in gravel-bed rivers, *Earth Surface Processes and Landforms*, 26(13), 1395-1408, doi:10.1002/esp.301

Wilcock, P. R., and J. C. Crowe (2003), Surface-based transport model for mixed-size sediment, *Journal of Hydraulic Engineering*, *129*(2), 120-128.

Wilcock, P. R., S. T. Kenworthy, and J. C. Crowe (2001), Experimental study of the transport of mixed sand and gravel, *Water Resources Research*, *37*(12), 3349-3358, doi:10.1029/2001wr000683.

Wilcock, P. R., J. Pitlick, and Y. Cui (2009), Sediment transport primer: estimating bed-material transport in gravel-bed rivers, *General Technical Report RMRS-GTR-226*, 78 pp., United States Department of Agriculture, Forest Service, Rocky Mountain Research Station, Fort Collins, CO.

Wright, N. G. (2005), Introduction to Numerical Methods for Fluid Flow, in Computational Fluid Dynamics, edited by P. Bates, S. Lane and R. Ferguson, pp. 147-168, John Wiley & Sons, Ltd, Chichester.

Wu, W. (2008), Computational river dynamics, 494 pp., Taylor & Francis, London.

Figure S1. Comparison between surveyed and predicted cross section elevations for numerical scheme sensitivity analysis. Pre- and post-storm surveys are labelled DEM_1_ and DEM_2_ respectively. Cross sections are located across areas of pertinent morphological change, as indicated on Figure 1h.

**Figure S2.** Sediment budgets for hydraulic sensitivity analysis for (a) Experiment 2a, (b) Experiment 2b, (c) Experiment 2c and (d) Experiment 2d. The shaded area on the histogram shows observed morphological change and the lines show model predictions. k_s_ is Nikuradse roughness length. ν_H_ is horizontal eddy viscosity. Q is discharge.


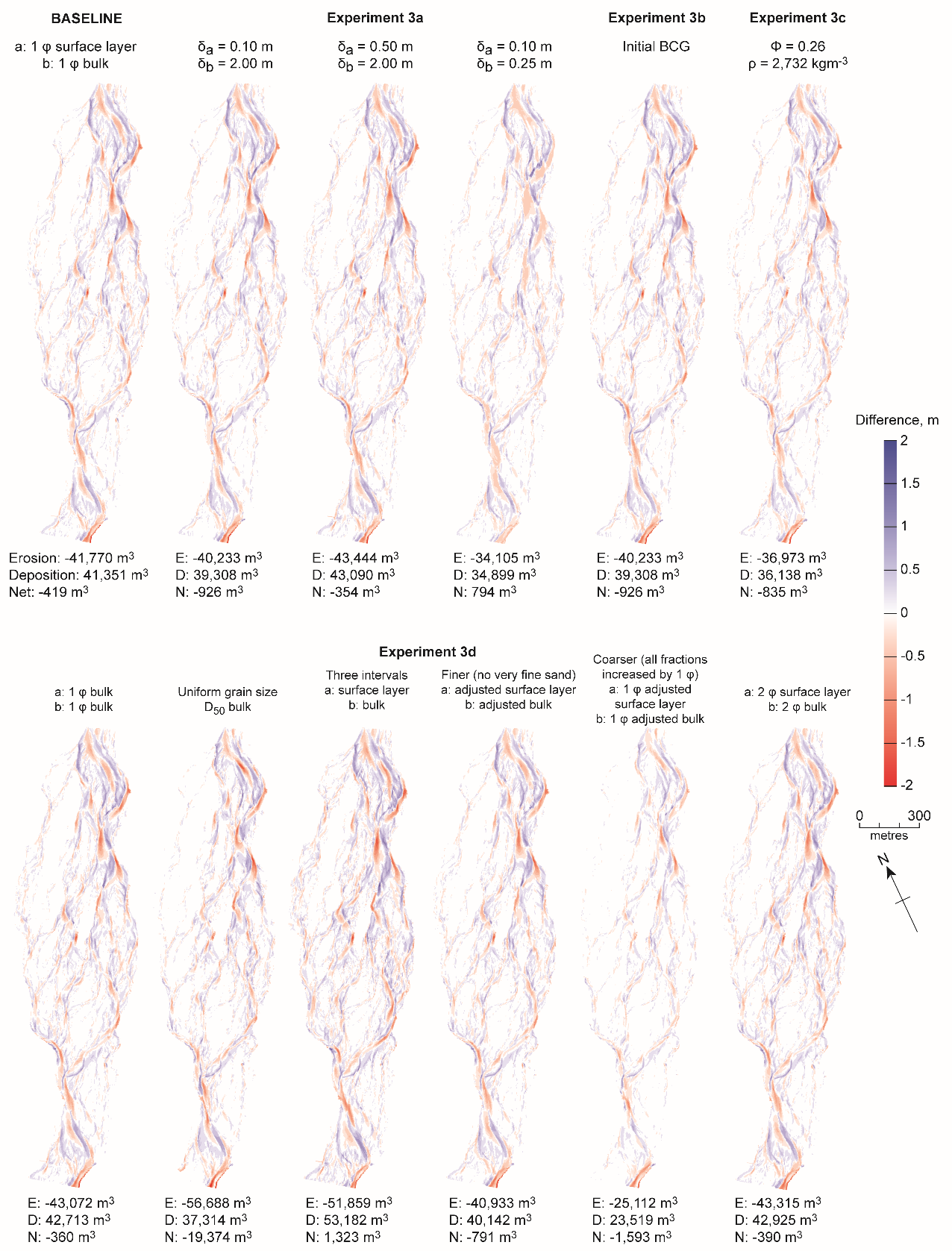


Figure S3. DoDs for bed composition sensitivity analysis (Experiment 3). δa is active layer. δu is under layer. BCG is bed composition generation. Φ is porosity. ρ is density. a is active layer. u is under layer. φ is grain size interval (i.e. 1 φ refers to a simulations with multiple grain sizes with 1 φ size divisions; 2 φ refers to a simulations with multiple grain sizes with 2 φ size divisions).

Figure S4. Sediment budgets for bed composition sensitivity analysis for (a) Experiment 3a, (b) Experiment 3b, (c) Experiment 3c and (d) Experiment 3d. The shaded area on the histogram shows observed morphological change and the lines show model predictions. Surface layer is abbreviated to SL. δa is active layer. δu is under layer. BCG is bed composition generation. Φ is porosity. ρ is density. *φ* is Krumbein grain size phi interval.


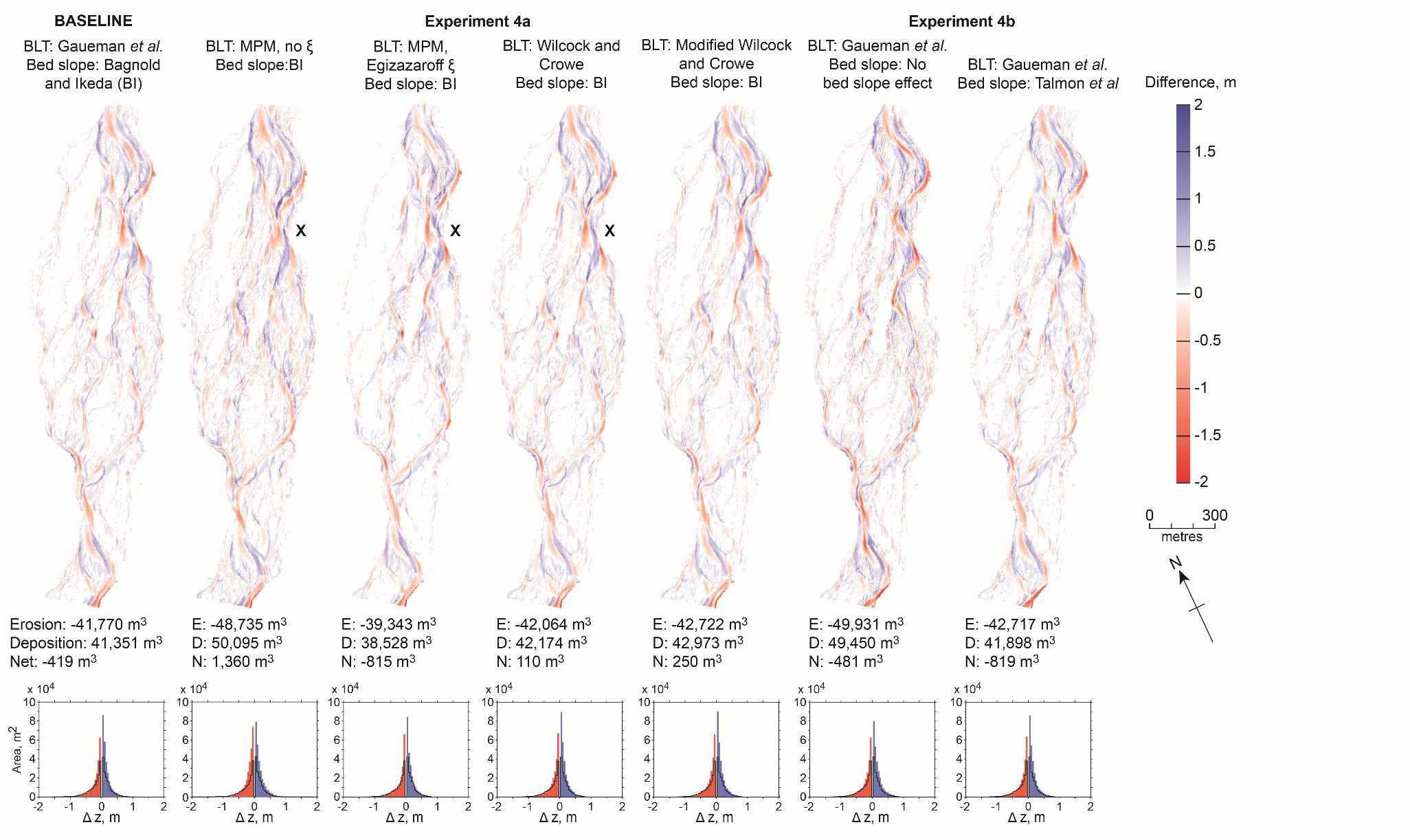


Figure S5. DoDs for bedload transport sensitivity analysis (Experiments 4a and b). MPM is Meyer-Peter and Müller. ξ is hiding and protrusion. BI is Bagnold and Ikeda. Letters identify areas of interest that are discussed in the text.


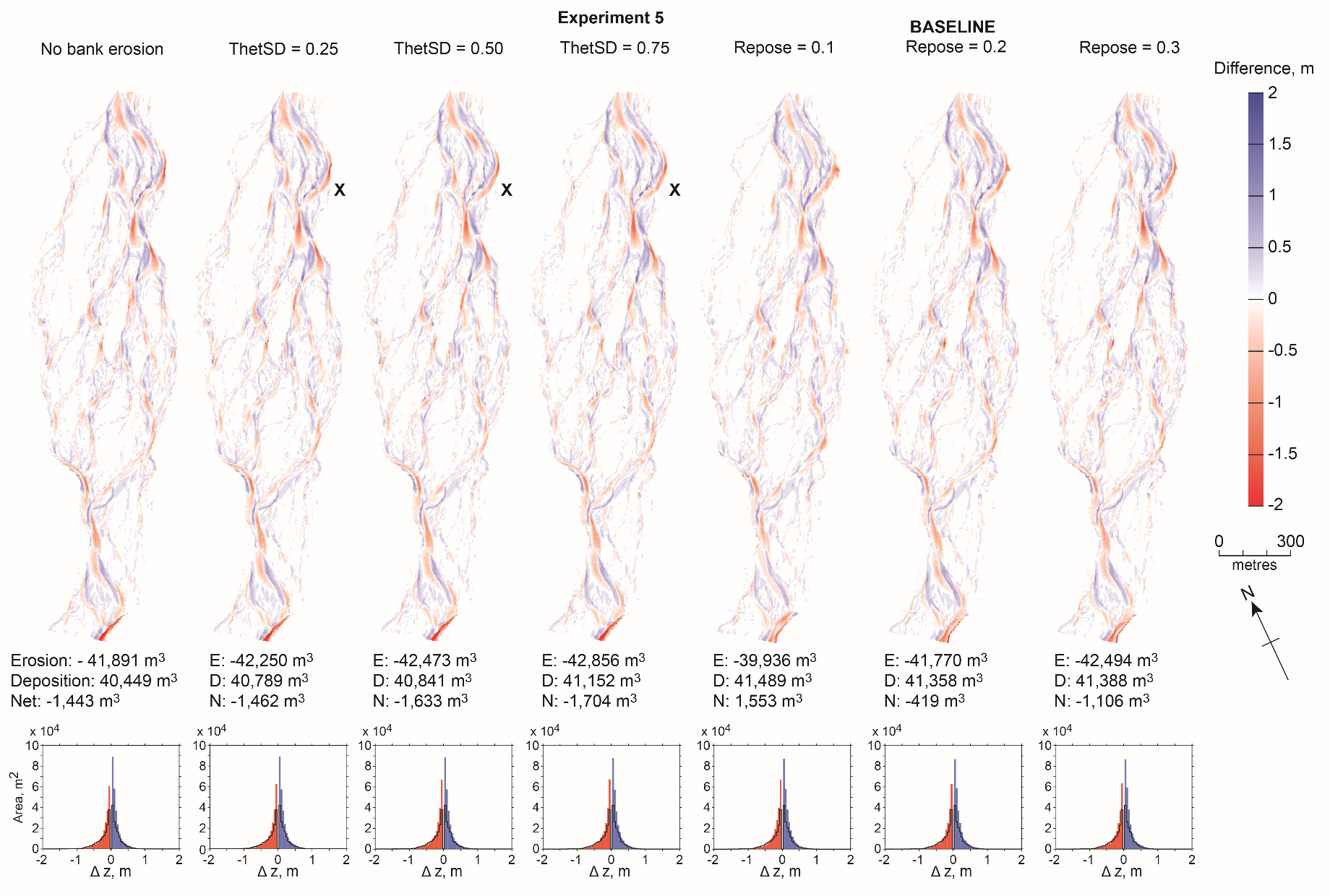


Figure S6. DoDs for bank erosion sensitivity analysis (Experiment 5). Letters identify areas of interest that are discussed in the text.

Figure S7. Sediment budgets for bank erosion sensitivity analysis sensitivity analysis (Experiment 5). The shaded area on the histogram shows observed morphological change and the lines show model predictions.


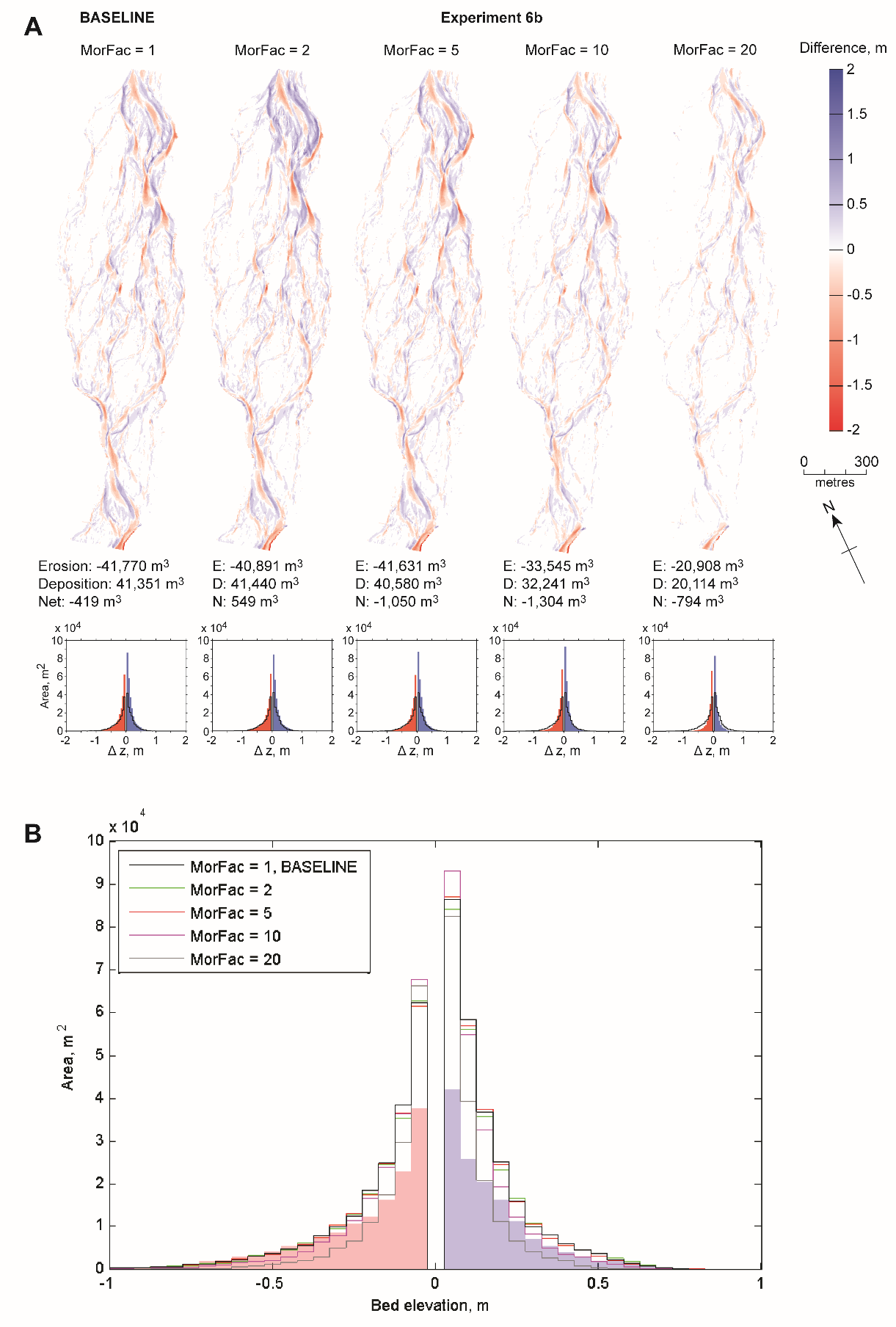

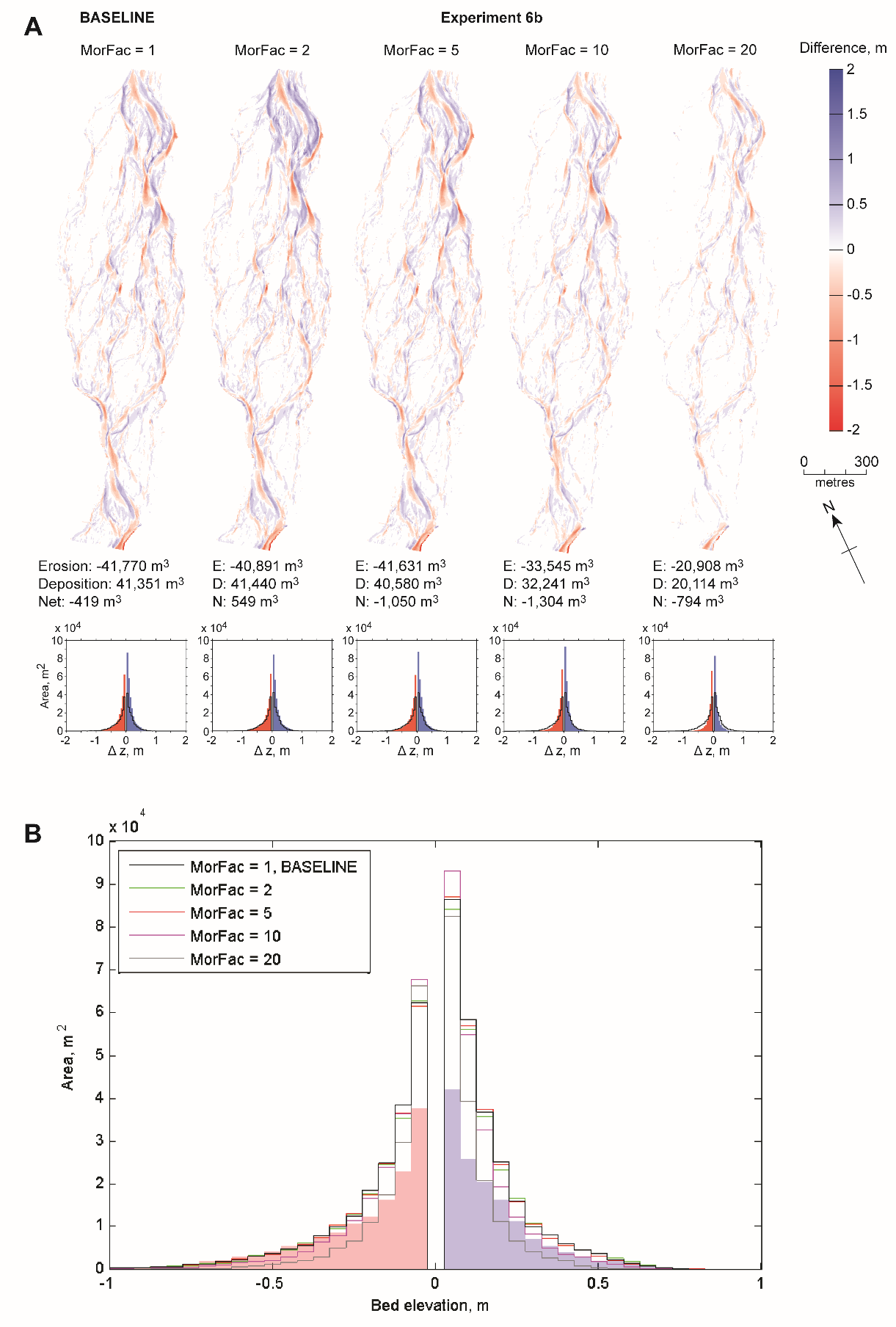


Figure S8. (a) DoDs for frequency of morphological calculations sensitivity analysis (Experiment 6). (b) Sediment budgets for frequency of morphological calculations sensitivity analysis (Experiment 6). The shaded area on the histogram shows observed morphological change and the lines show model predictions.
